# Anlotinib Benefits the *α*PDL1 Immunotherapy by Activating ROS/JNK/AP-1 Pathway to Upregulate PDL1 Expression in Colorectal Cancer

**DOI:** 10.1155/2022/8965903

**Published:** 2022-10-04

**Authors:** Bixian Luo, Shun Zhang, Dan Tan, Xinbo Yu, Jianwei Lin, Mingliang Wang

**Affiliations:** ^1^Department of General Surgery, Ruijin Hospital, Shanghai Jiao Tong University School of Medicine, Shanghai, China; ^2^Department of Urology, Xinhua Hospital, School of Medicine, Shanghai Jiao Tong University, Shanghai, China; ^3^Department of General Surgery, Ruijin Hospital Luwan Branch, Shanghai Jiao Tong University School of Medicine, Shanghai, China; ^4^Department of Urology, Ruijin Hospital, Shanghai Jiao Tong University School of Medicine, Shanghai, China

## Abstract

Colorectal cancer (CRC) is one of the prevalent malignant tumors. This study is aimed at evaluating the mechanism of anlotinib (anlo) on tumor microenvironment (TME) in CRC, and its effects in combination with immune checkpoint inhibitors (ICIs) therapy. Firstly, MC38 and CT26 cells were both exposed to different gradient concentrations of anlo for 72 h, to investigate the cell viability and synergetic therapy efficacy with ICIs by CCK8. The results showed that anlo could obviously inhibit cell growth and showed no synergistic efficacy therapy in combination with *α*PDL1 *in vitro*. Then, we found the upregulation of programmed cell death ligand 1(PDL1) expression both *in vitro* and *in vivo* after anlo treatment. *In vivo*, anlo could enhance the percentage of natural killer (NK) cells and M1 macrophage cells and decrease the percentage of M2 macrophage cells in TME. Moreover, we explored the mechanism and we proved that anlo could activate reactive oxygen species (ROS)/c-Jun N-terminal kinase (JNK)/activator protein-1 (AP-1) signaling pathway to increase the expression levels of PDL1, IFN-*α*/*β*/*γ*, and CXCL2 in two cell lines *in vitro*. We also proved that anlo had synergistic effects with ICIs *in vivo*. Finally, it could also increase the mRNA and protein PDL1 expression levels in human cell lines, which was consistent with mouse CRC cell lines. However, there are still a few limitations. On one hand, the ROS/JNK/AP-1 pathway needs to be proved whether it can be activated in human cell lines. On the other hand, the mechanism behind ROS promoting phosphorylation of JNK needs to be explored.

## 1. Introduction

CRC is one of the most common malignant tumors and poses a serious threat to human public health worldwide. According to 2021 cancer statistics of American Cancer Society, CRC ranks third both in the new incidence and in the death spectrum of malignant tumors, and there are 149,500 new cases and about 52,980 deaths in the United States [1]. In 2015, there are an estimated 376,300 new cases and 191,000 deaths of CRC in China [2]. Clinical epidemiological investigation results show that the relative 5-year survival rate of CRC patients is 65%, while the 5-year survival rate of patients with advanced stage IV CRC is only 12% [3]. Thus, it is essential to prolong the overall survival time of CRC patients.

As reported, immunotherapy has become a new trend of controlling tumor initiation, growth, and progression [4, 5]. Unlike traditional tumor treatment methods (surgery, radiotherapy, and chemotherapy) aimed at targeting and killing tumor cells, the core of immunotherapy is to mobilize and activate the patient's own immune system to control and kill tumor cells, so as to achieve the purpose of suppression or cure of cancer. PDL1 (CD274) is frequently observed in tumor cells, and PD1 (CD279) is expressed in immune cells (T lymphocytes, macrophages, and others). The combination of both PD1 and PDL1 can induce lymphocyte inactivation, and tumor cells cannot be recognized and killed by immune cells [6, 7]. In clinical trials, a variety of PDL1 and PD1 immunohistochemical assays to assess PDL1 and PD1 expression on tumor and immune cells are used as potential biomarkers for predicting immunotherapy responses [8]. In CRC, patients who have microsatellite instability-High (MSI-H) and mismatch repair defection (dMMR) in tumor genetic phenotypes have more positive responses to PD1/PDL1 inhibitor immune treatment [9, 10]. Up to now, the FDA has approved pembrolizumab (PD1 inhibitor) for the first-line MSI-H/dMMR mCRC treatment [11]. Although PD1/PDL1 inhibitors contribute to a new therapeutic model for CRC, only a small percentage of advanced/metastatic CRC patients have a survival benefit from PD-1/PDL1 inhibitor treatment [12]. Therefore, how to improve the effectiveness of immunotherapy for CRC is the key scientific problem to be solved in this study.

Anlo is a multitarget tyrosine kinase inhibitor (TKI) and can simultaneously inhibit VEGFR2/3, FGFR1-4, and PDGFR*α*/*β* [13]. In the aspect of CRC, Jia et al. proved that anlo could inhibit the growth of CRC by inactivating VEGFR/JAK2/STAT3 signaling pathway [14]. Lan et al. also found that anlo could reverse multiple drug resistant CRC cells by antagonizing PI3K/AKT axis [15]. A recent study proved that anlo increased ROS and induced apoptosis via activation of endoplasmic reticulum stress in pancreatic cancer [16]. In recent clinical research of anlo, an ALTER0703 phase III study showed that in patients with advanced CRC who had failed second-line chemotherapy, anlo could significantly improve progression-free survival (PFS) and was safe and well tolerated for patients [17]. Meanwhile, a multicenter clinic phase II study proved anlo combined with capecitabine and oxaliplatin showed considerable objective response rate (ORR), PFS, and duration of response (DOR) in mCRC with manageable toxicity profiles [18]. In a subcutaneous tumor-bearing model of lung cancer LLC cells, anlo increased NK cell infiltration, enhanced NK cell and CD4+ T cell to secret more IFN-*γ*, and increased mature antigen-presenting cells [19]. In the mouse model of neuroblastoma, anlo significantly inhibited tumor growth and effectively prevented systemic immunosuppression by promoting tumor vascular normalization and immune cell infiltration. In addition, anlo combined with PD1 checkpoint inhibitors counterbalanced immunity induced by PDL1 upregulation after monotherapy [20]. In B16 mouse melanoma and MC38 mouse colon cancer models, anlo could reduce the expression of PDL1 in vascular endothelial cells, thereby increasing the ratio of CD8+ T cells in tumors and increasing the invasion of CTL into tumors [21].

Targeting these immune checkpoints to improve antitumor immunity is currently one of the most promising therapeutic strategies in CRC as well as in a large variety of other malignancies. Monotherapy with ICIs has recently shown striking results in clinical trials in different tumors. Anlo combining other agents targeted to different biological receptors or pathways (such as PD1/PDL1 pathway) may be a new treatment strategy to improve the therapeutic efficacy in CRC. In this study, we used both *in vitro* and *in vivo* ways to explore the antitumor efficacy of anlo combined with ICIs. In a word, the current study was aimed at determining whether the combination of anlo and ICIs had a better efficacy treatment and what was the possible underlying mechanisms behind it, so as to provide a new strategy for clinical treatment.

## 2. Method

### 2.1. Cell Lines and Reagents

Both mouse (MC38 and CT26) and human (HCT-116 and RKO) CRC cell lines were obtained from Institute of Immunology, Shanghai, China. All of these cells were grown in DMEM medium with 10% fetal bovine serum (Gibco Catalog No. 10100147). Cells were incubated at 37°C in a humidified atmosphere with 5% CO_2_. Anlo was obtained from CTTQ (Chia Tai Tian Qing) (pharmaceutical group, Nanjing, China). JNK1/2 inhibitor was obtained from MedChemExpress (Shanghai, China, #129-56-6), mouse CXCL2/MIP-2 ELISA Kit from MULTI SCIENCES (Shanghai, China, #EK2142), and mouse-*α*PDL1 from bioXcell (#BE0361).

### 2.2. Proliferation Assay

Before the experiments, cells were seeded into 96-well plates at a density of 2000 cells per well and cultured for 24 h. At the end of treatments of anlo or *α*PDL1, cell proliferation assays were performed using Cell Counting Kit-8 (DOJINDO, Tokyo, Japan) according to the manufacturer's instructions. In brief, each well was added with 110 *μ*L working solution buffer (containing 100 *μ*L DMEM and 10 *μ*L CCK8 reagent). After 4 h of further incubation (37°C, 5% CO_2_), the absorbance was determined with a microplate reader (Bio Tek, Vermont, USA) at the wavelength of 450 nm.

### 2.3. Quantitative Real-Time PCR

The total RNA was extracted from cells using Trizol (Invitrogen, Carlsbad, CA, USA). For cDNA synthesis, the reverse transcriptional reaction was using PrimeScript™ RT reagent Kit (Takara Bio, Tokyo, Japan) in a 20 *μ*L reaction system. Quantitative real-time PCR was performed using TB Green™ Premix Ex Taq™ II (Takara Bio, Tokyo, Japan) according to the manufacturer's instructions. The primers used for PDL1 are shown in Supplementary Table 1. We used *β*-actin primers for internal control. Gene expression was normalized to *β*-actin according to the cycle threshold (2^−*Δ*CT^) method.

### 2.4. Western Blot Analysis

Cells were harvested and lysed in RIPA buffer supplemented with protease inhibitor cocktail (Epizyme Biotech). Protein concentration was determined with a BCA protein assay kit (Takara Bio, Tokyo, Japan). Protein samples were separated by electrophoresis on sodium dodecyl sulfate-polyacrylamide gel electrophoresis (SDS–PAGE) gels and transferred to a polyvinylidene fluoride (PVDF) membrane. After blocking with skim milk for 1 hour in TBST, the membranes were incubated with the primary antibodies included anti-PDL1 (#1-76769, Novus, USA, 1 : 1000), anti-PDL1 (#DF6526, affinity, 1 : 1000), anti-HSP90 (#4877, CST, Boston, MA, USA, 1 : 1000), anti-IFN*α* (#DF6086, affinity, 1 : 1000), anti-IFN*β* (#ab218229, Abcam, 1 : 1000), Anti-IFN*γ* (#DF6045, affinity, 1 : 1000), anti-JNK2 (#9258, CST, 1 : 1000), P-JNK (#4668, CST, 1 : 1000), P-c-fos (#5348, CST, 1 : 1000), and P-c-jun (#2361, CST, 1 : 1000) at 4°C overnight. The membranes were washed three times with TBST, 10 min each time, and subsequently incubated with horseradish peroxidase- (HRP-) conjugated secondary antibody (#7074, CST, Boston, MA, USA, 1 : 5000) for 1 h. Protein bands were visualized using the ECL Prime Western Blotting Detection System (32209, Thermo Fisher Scientific, Waltham, MA, USA).

### 2.5. Immunofluorescence Analysis

After treatment, cells were fixed in 4% formaldehyde at room temperature for 24 h and blocked with 5% goat serum for 60 min. Primary antibody Ki67 (#ab15580, Abcam, 1 : 200) was incubated at 4°C overnight. The following day, the cells were washed with PBS and incubated with the secondary antibody Alexa Fluor 488 anti-rabbit (#4412, CST, 1 : 500) and Alexa Fluor 647 anti-rabbit (#4414, CST, 1 : 50) for 2 h at room temperature. Nuclei were stained with DAPI. Images were obtained with a fluorescence microscope (Eclipse 80i; Nikon Corporation).

### 2.6. Detection of ROS

For cellular ROS detection, a ROS assay kit (Beyotime Institute of Biotechnology) was used. After treatment, cells were incubated with 10 *μ*M dichlorodihydrofluorescein diacetate probe at 37°C for 20 min. Finally, the intracellular ROS levels were detected using a flow cytometer.

### 2.7. Animal Studies

Six to eight-week-old female C57BL/6J and Balb/c mice weighing 18-20 g obtained from the animal experiment center (JiHui Laboratory Animal Corp. Ltd, Shanghai, China) were used in all experiments. Mice had free access to food and water and were housed and maintained at Ruijin Hospital Laboratory Animal Resource Facility. Animals were assessed daily by veterinary staff at our institution and by qualified investigators in our group. All animal procedures were approved by the Animal Ethics Committee of Ruijin Hospital and in conformity to the Guide for Care and Use of Laboratory Animals.

Each 1.5 million number of MC38 and CT26 cells was separately injected into the flanks of both C57BL/6 and Balb/c mice (6-8 weeks). Anlo (1.5 mg/kg) was initiated 8 days after tumor cell inoculation. *α*PDL1 treatment was injected at the 8th day, 11th day, and 13th day. Tumor volume was measured as follows: (length × width^2^)/2. At the 15th day, the tumor-bearing mice were sacrificed and tumors were surgically removed. The tumors were weighed, processed for IHC staining, and harvested for analysis.

### 2.8. Preparation of Single Cell Suspensions and Flow Cytometry

The tumors were weighed about 0.3 g to digest in DMEM with dissociative enzyme from using mouse tumor dissociation kit (Miltenyi Biotec, #130-096-730) at 200 rpm for 45 min at 37°C. The cell suspensions were filtered through sieves. Red Blood Cell Lysis Buffer (Beyotime Biotec, Shanghai, China) was used to lyse erythrocytes. For surface markers, single cells were stained with the following anti-mouse mAbs for 30 min at 4°C to stain macrophage, NK and T cell panel: CD45 (APC-Cy7, BD, #557659), F4/80 (BV421, BD, #565411), CD86 (PE, BD, #551396), PDL1 (BV711, BD, #563369), CD11B (FITC, BD, #561684), CD3 (BV711, BD, #563123), CD8 (PE-CY7, BD, #552877), NKp46 (BV421, Biolegend, #137612), NK1.1 (BV650, BD, #564143), and Fixable Viability Stain (BV510, BD, #564406). After surface staining, the cells were fixed, permeabilized, and stained with the following anti-mouse mAbs for 30 min at 4°C: CD206 (BV650, Biolegend, #103864), IFN-*γ* (FITC, BD, #554422), IL-2 (BV605, BD, #563911), IL-17A (PE, BD, #561020), and TNF-*α* (APC, Biolegend, #506308). Data were acquired using a BD LSRFortessa™ X-20 instrument (BD Biosciences, San Jose, CA, USA) and analyzed using FlowJo software (Tree Star Inc., Ashland, OR, USA).

### 2.9. Immunohistochemistry (IHC) Analysis

The paraffin-embedded mouse tumor tissue sections were deparaffinized, and after antigen retrieval, the slides were stained with mouse Ab against PDL1 (#ab238697, 1 : 500). The number of PDL1+ cells was evaluated in 5 fields per section (original magnification, X400) by Image-Pro Plus 6.0.

### 2.10. RNA Sequencing Analysis

Total RNA was extracted from CT26 tumor tissues using an AllPrep DNA/RNA/miRNA Universal Kit (Cat No 80224; Qiagen) and subjected to the transcriptome assay (Shanghai Tsingke Biotech). The heatmap was generated by using the “pheatmap” package of R language based on differentially expressed genes.

### 2.11. Statistical Analysis

Statistical analysis was performed using GraphPad Prism8 software (GraphPad Software, San Diego, CA, USA). Data are expressed as the means ± standard deviations (SD). The differences between sets of data were analyzed with two-tailed Student's *t* test and two-way ANOVA. *p* < 0.05 was considered statistically significant.

## 3. Results

### 3.1. Anlo in Combination with *α*PDL1 Had No Extra Therapy Efficacy In Vitro Study of Both MC38 and CT26 Cells

**Figure 1:**
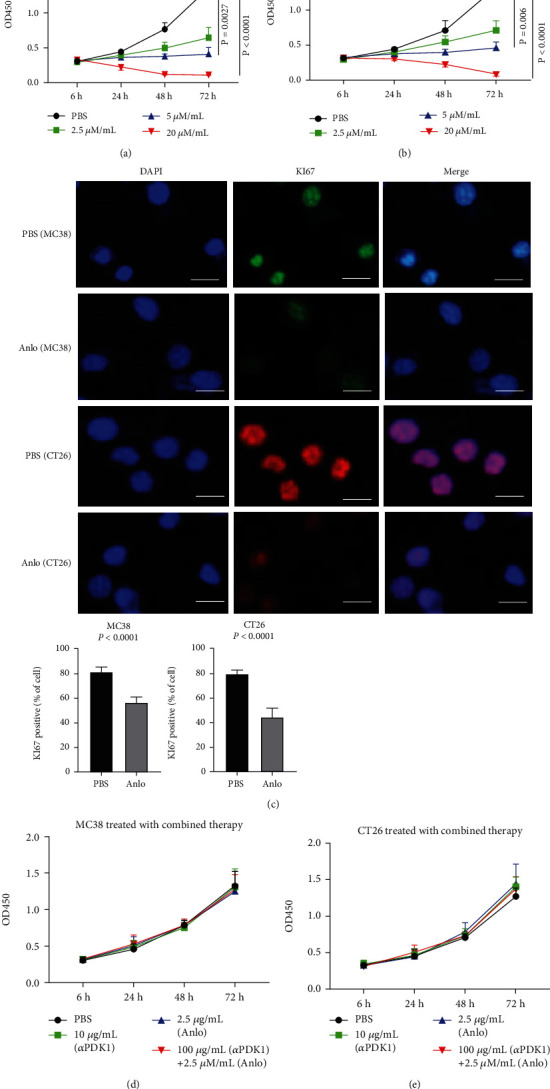
Cell proliferation assay analyzed by CCK8. (a) MC38 cells were treated with anlo (2.5 *μ*M/mL, 5 *μ*M/mL, and 20 *μ*M/mL) for 6 to 72 h. (b) CT26 cells were treated with anlo (2.5 *μ*M/mL, 5 *μ*M/mL, and 20 *μ*M/mL) for 6 to 72 h. (c) Representative images of immunofluorescence were observed by fluorescence microscopy, which confirmed the expression of KI67, and quantification of the results. (d) MC38 cells were assigned for four groups, which were PBS, anlo (2.5 *μ*M/mL), *α*PDL1 (10 *μ*g/mL), and combination group (anlo and *α*PDL1) for 6 to 72 h. (e) CT26 cells were assigned for four groups, which were PBS, anlo (2.5 *μ*M/mL), *α*PDL1 (10 *μ*g/mL), and combination group (anlo and *α*PDL1) for 6 to 72 h.

We first wanted to determine whether anlo could inhibit cell proliferation at four different concentrations: 0 *μ*M/mL, 2.5 *μ*M/mL, 5 *μ*M/mL, and 20 *μ*M/mL. CCK8 results showed that the viability of both MC38 and CT26 was remarkably suppressed in a dose-dependent manner (PBS vs. 20 *μ*M/mL, *p* < 0.0001). In the highest drug concentration group (20 *μ*M/mL), they then gradually stopped growing, and eventually, they died in a time-dependent manner (Figures 1(a) and 1(b)). The results of Immunofluorescence analysis consolidated its tumor-suppressive ability because anlo could reduce the expression of KI67 (Figure 1(c)). Then, we treated these with different concentrations of *α*PDL1 (0, 1, 10, and 100 *μ*g/mL) for 72 h. Unsurprisingly, there was no any inhibition at four different concentrations of *α*PDL1 **(**Supplementary Figures 1(a) and 1(b)). Then, 2.5 *μ*M/mL anlo with 10 *μ*g/mL *α*PDL1 was designed to coculture for 72 h. Anlo in combination with *α*PDL1 did not show any additional inhibition when compared with anlo monotherapy (Figures 1(d) and 1(e)).

### 3.2. Anlo Had the Same Effects on the Expression of PDL1 In Vivo and In Vitro

**Figure 2:**
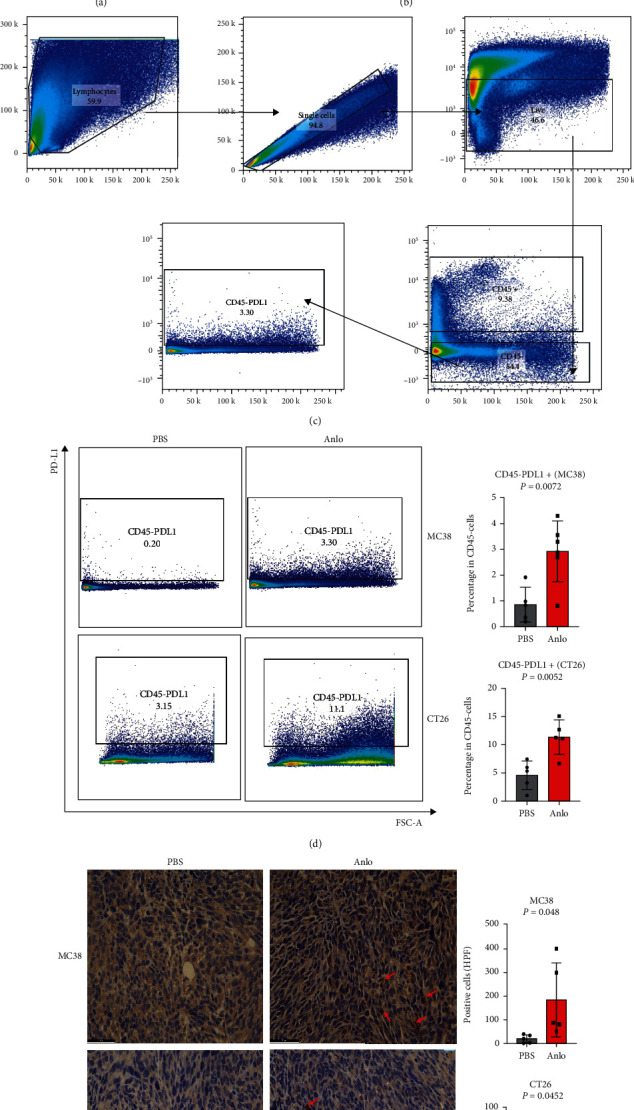
Anlo elevated the expression of PDL1 *in vivo* and *in vitro*. (a) PDL1 mRNA expression was elevated after anlo treatment (2.5 *μ*M/mL) in MC38 and CT26 cells. (b) PDL1 protein expression was elevated after anlo treatment (2.5 *μ*M/mL, 5 *μ*M/mL) in MC38 and CT26 cells. (c) Gating strategy of flow analysis. (d) The percentage of PDL1 in CD45 cells of both MC38 and CT26 cell line tumor-bearing mouse models after anlo treatment. (e) Immunohistochemistry results of PDL1 in MC38 and CT26 tumor-bearing mice after anlo treatment; the red arrows represent the positive expression of PDL1 protein of both MC38 and CT26 tumors.

Results of RT-PCR and western blot showed that PDL1 was elevated in both mRNA and protein levels *in vitro* (Figures 2(a) and 2(b)). Then, we analyzed PDL1 expression in tumor-bearing mouse models. Gating strategy of flow analysis is shown in Figure 2(c). The results demonstrated that the percentage of PDL1 in CD45-cell of both MC38 and CT26 cell lines tumor-bearing mouse models was dramatically overexpressed (MC38 mouse model: *p* = 0.0072, CT26 mouse model: *p* = 0.0052) (Figure 2(d)). Immunohistochemical (IHC) staining of PDL1 of tumor tissues consolidated the conclusions above (Figure 2(e)).

### 3.3. Anlo Boosted Tumor-Infiltrating NK Cells

**Figure 3:**
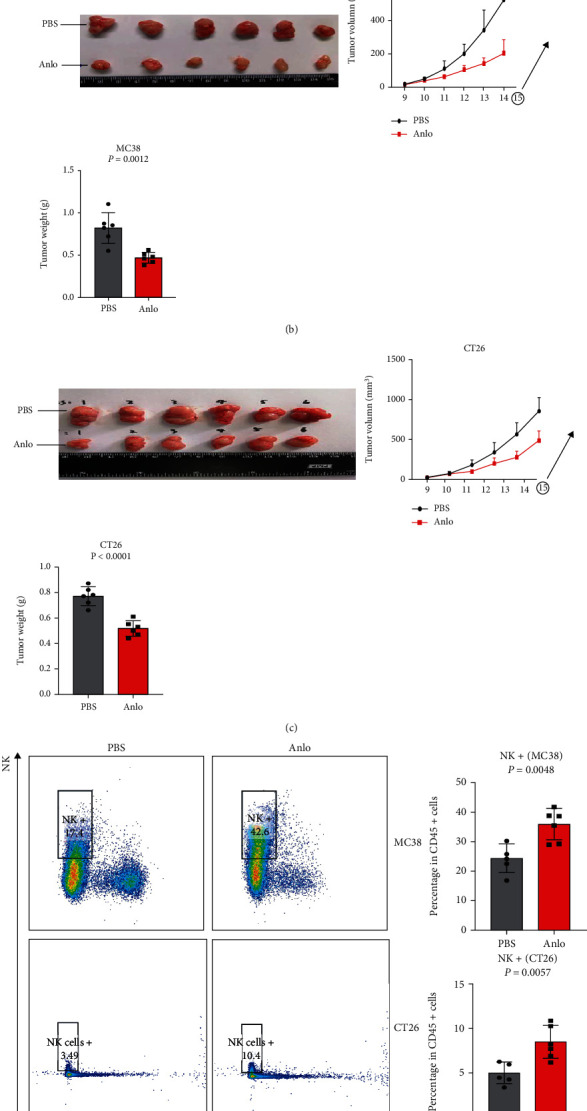
Anlo boosted tumor-infiltrating NK cells. (a) MC38/CT26 cells were injected subcutaneously into mice, and after a week, mice were treated with PBS or anlo for a week (*n* = 6). (b) Gross appearance, tumor volume, and tumor weight of MC38 tumor. (c) Gross appearance, tumor volume, and tumor weight of CT26 tumor. (d) Flow cytometric analysis of MC38/CT26 tumor-infiltrating NK cells.

Many published studies have proved that anlo could reprogram the immunosuppressive tumor microenvironment. In our study, it deserved to be investigated whether anlo played an immune-promoting role in CRC. The timeline of the animal experiments was shown in (Figure 3(a)). As shown in (Figures 3(b) and 3(c)), anlo exhibited inhibitory effect on the MC38 and CT26 tumor weight and volume. Then, the tumor immune microenvironment was analyzed and the gating strategy of T and NK+ cells is shown in Supplementary Figure 2. It was noticed that NK cells were elevated in both MC38 and CT26 mouse models (MC38 mouse model: *p* = 0.0048, CT26 mouse model: *p* = 0.0057) (Figure 3(d)) and anlo did not increase the functions of NK+ cells (Supplementary Figures 3(a)–3(h)). Separately speaking, even if anlo did not change the number of CD4+ and CD8+ T cells (Supplementary Figures 4(a) and 4(b)), it could enhance the function of CD8+ T cells to secret more IFN-*γ* and TNF-*α* in the MC38 mouse model (Supplementary Figures 4(c) and 4(d)). In the CT26 mouse model, anlo could increase the number of CD4+ cells but not CD8+ cells in the CT26 tumor-bearing mouse model (Supplementary Figures 4(e) and 4(f)).

### 3.4. Anlo Activated M1 Macrophage Cells

**Figure 4:**
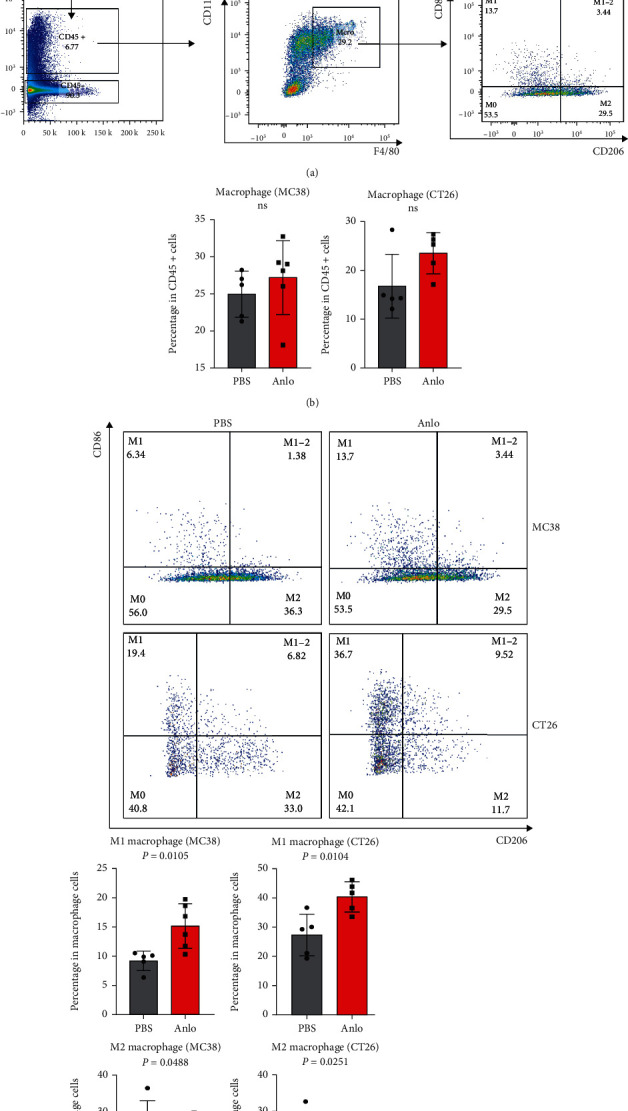
Anlo activated M1 macrophage cells. (a) Gating strategy of flow analysis for macrophage cells. (b) The effect of anlo on intratumoral macrophage cells. (c) Flow cytometric analysis of MC38/CT26 tumor-infiltrating M1 and M2 macrophage cells.

How to gate macrophage with its subtypes is presented in Figure 4(a), and the effects of anlo on macrophage cells were analyzed. It was observed that the intratumoral macrophage levels in the anlo groups remained not significant to that of the control groups (Figure 4(b)). However, we found that M1 microphage cells (CD86+CD206-) were elevated, and M2 microphage cells (CD86-CD206+) downregulated, in both two mouse models (Figure 4(c)). What is more, anlo could upregulate the expression of PD1 in both M1 and M2 macrophage cells in MC38, but not in CT26, mouse model (Supplementary Figures 5(a)–5(d)). Finally, we found that the upregulated expression of PDL1 of M2 macrophage cells in both mouse models (Supplementary Figures 5(e)–5(f)).

### 3.5. Anlo Increased the Expression of PDL1 by ROS/JNK/AP-1 Pathway In Vitro

**Figure 5:**
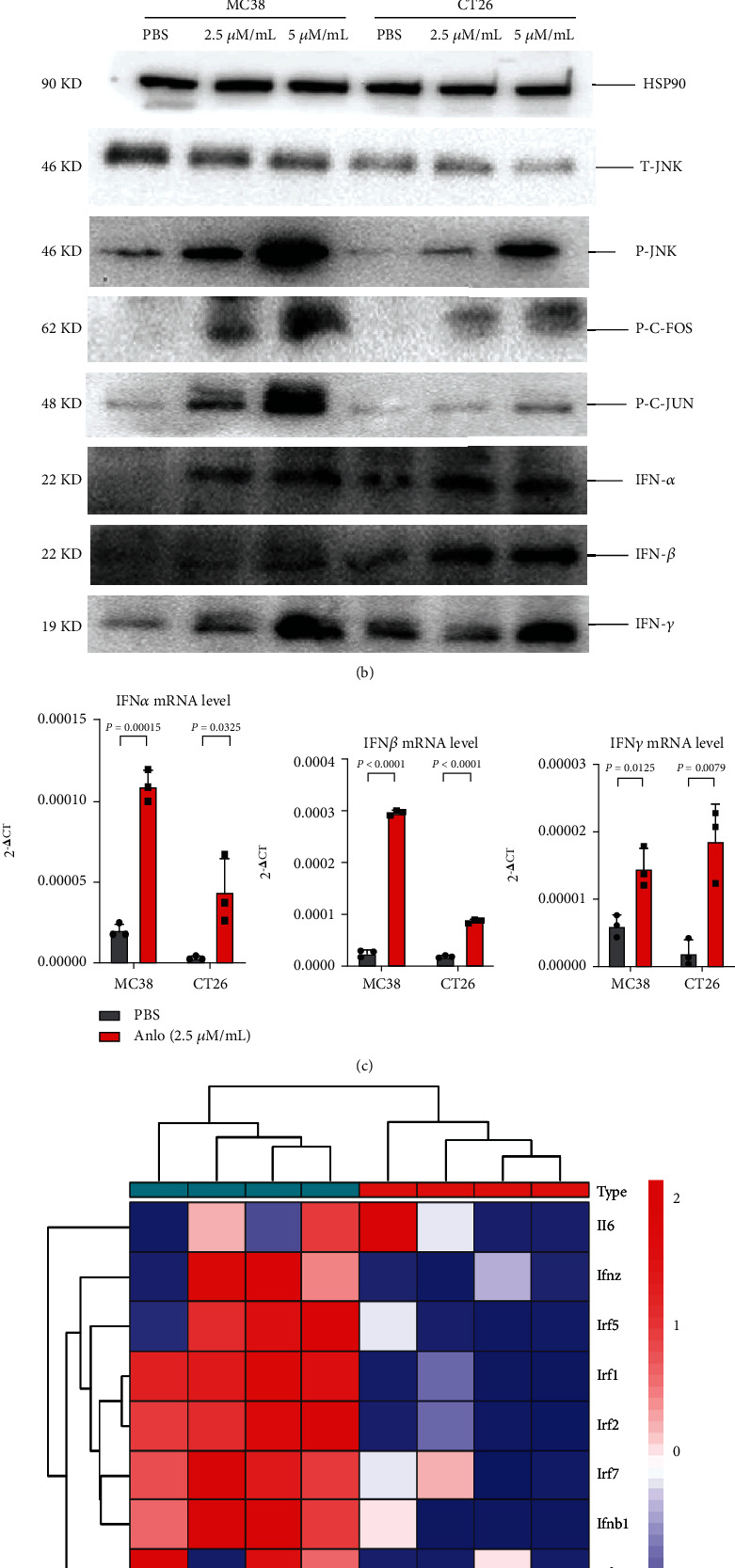
Anlo increased the expression of PDL1 by ROS/JNK/AP-1 pathway *in vitro*. (a) ROS production was determined and analyzed by flow cytometry. (b) Representative western blots of HSP90, T-JNK, P-JNK, P-C-FOS, P-C-JUN, IFN-*α*, IFN-*β*, and IFN-*γ*. (c) The expression of mRNA levels of IFN-*α*, IFN-*β*, and IFN-*γ*. (d) RNA sequence data analysis of CT26 tumor.

We continued to explore the underlying mechanisms of elevated expression of PDL1 after anlo treatment and found that anlo could enhance the ROS level in both cell lines (Figure 5(a)). We found that JNK/AP-1 pathway was activated by anlo, which could promote interferon responses and PDL1 expression (Figures 5(b) and 5(c)). RNA sequencing analysis of CT26 tumor consolidated the conclusions above (Figure 5(d)). Moreover, it was observed that CXCL2 was increased both in mRNA level and cell culture serum (Supplementary Figure 6(a)). Furthermore, we used the JNK inhibitor to inactivate the JNK/AP-1 pathway to find whether PDL1 expression and IFN-*α*/*β*/*γ* (IFN-*α*/*β* known as type I interferon, IFN-*γ* known as type II interferon) were influenced. The results showed that the expression of CXCL2, IFN-*β*/*γ*, and PDL1 did not change much compared with baseline expression levels (Supplementary Figures 6(b) and 6(c)).

### 3.6. Anlo Had Synergistic Antitumor Activity of PDL1 Blockade in CRC Mouse Models

**Figure 6:**
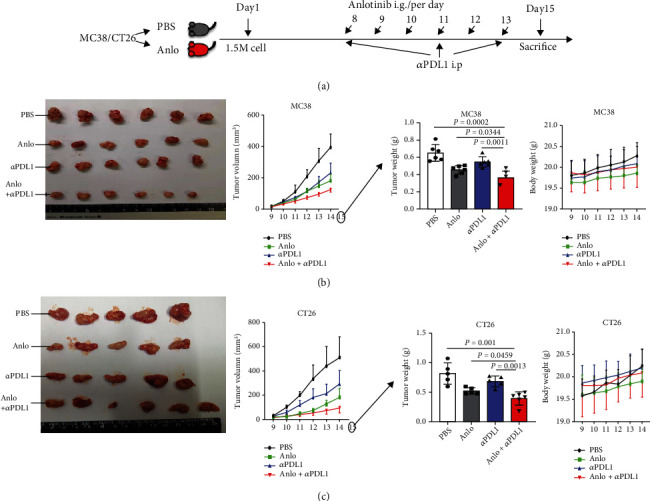
Anlo improved the anti-tumor activity of PDL1 blockade in CRC mouse models. (a) MC38/CT26 cells were injected subcutaneously into mice; after a week, mice were treated with PBS, anlo (six times), *α*PDL1 (three times), and combination therapy (anlo/six times and *α*PDL1/three times). (b) Gross appearance, tumor volume, tumor weight, and body weight of MC38 tumor. (c) Gross appearance, tumor volume, tumor weight, and body weight of CT26 tumor.

As mentioned above, after anlo treatment, the percentage of NK cells and M1 macrophage cells and PDL1 expression was elevated. Therefore, Then, it was tested whether anlo had a synergistic effect with *α*PDL1 in CRC mouse models. Anlo and *α*PDL1 given to MC38 and CT26-bearing mice were administered on the following plans: anlo treatment, followed by the administration of *α*PDL1 or PBS control started on day 08 (Figure 6(a)). We could find that no matter tumor weight or tumor volume, anlo plus *α*PDL1 treatment suppressed tumor growth more remarkably compared with the *α*PDL1 or anlo monotherapy (MC38 mouse model (anlo+*α*PDL1 vs. *α*PDL1/anlo/PBS): *p* = 0.0011/0.034/0.0002, CT26 mouse model (anlo+*α*PDL1 vs *α*PDL1/anlo/PBS): *p* = 0.0013/0.0459/0.001), and the body weight measured among all groups was not statistically significant, proving that there was no obvious toxicity (Figures 6(b) and 6(c)).

### 3.7. Anlo Elevated PDL1 Expression of Human CRC Cell Lines in a Concentration-Dependent Manner

**Figure 7:**
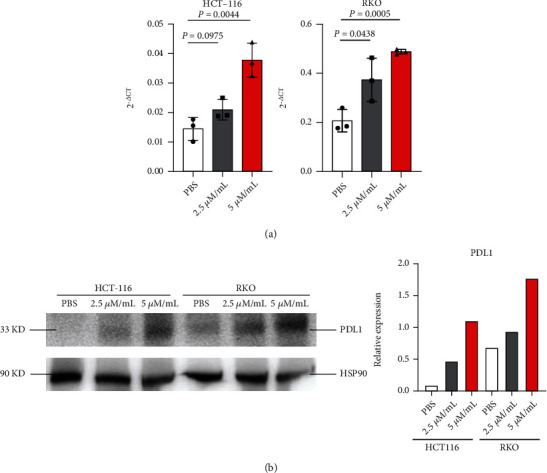
Anlo elevated PDL1 expression of human CRC cell lines in a concentration-dependent manner. (a) mRNA expression levels of PDL1 in HCT-116 and RKO cells treated with PBS, 2.5, and 5 *μ*M/mL anlo. (b) Protein expression levels of PDL1 in HCT-116 and RKO cells were proved by western blots and the quantification of the blotting results.

The conclusions above showed that anlo could increase PDL1 expression both *in vivo and in vitro* using mouse CRC cell lines and its tumor-bearing mouse models. We finally wanted to explore whether anlo could elevate PDL1 expression on human CRC cell lines. The results of RT-PCR and western blot of HCT-116 and RKO showed that anlo could induce human CRC cell lines to overexpress PDL1 (Figures 7(a) and 7(b)).

## 4. Discussion

Anlo, a novel TKI, was shown a favorable efficacy and acceptable safety in the treatment of mCRC [17, 18]. The combination of ICIs and targeted therapy serves as a potential novel therapeutic approach for many cancers. However, the lack of systematic representations of how anlo affects TME makes it sense to explore the underlying mechanisms after treatment of anlo in CRC, providing theoretical evidence for the combined use of ICIs with anlo for the clinic.

The PD1/PDL1 pathway is one of the most important signaling pathways mediating immunosuppression and tumor immune escape. When PD1-mediated inhibitory signals are activated by its ligand (PDL1), the functions and cell survival proteins of immune cells are decreased, which means their abilities to clear tumor cells are impaired [22, 23]. The collective clinical evidence to date suggests that ICIs are most effective in inflamed tumors as characterized by tumor PD-L1 expression, high CD8+ T cell density, or the presence of a strong IFN-*γ* cytolytic T cell signature [24–26]. In mCRC, a clinic trial KEYNOTE-028 based on PDL1 expression to explore the response to ICIs showed that PD-L1 expression ≥ 1% was evaluated for responses to pembrolizumab [27]. Therefore, blocking PD1/PDL1 therapy is a promising treatment strategy by promoting killer cells against tumors. In our study, we found that anlo could increase PDL1 expression both *in vivo* and *in vitro*, which predicted its combination efficacy with *α*PDL1 therapy to improve CRC outcomes. NK cells are key effectors of anti-tumor immune responses and major targets of ICIs. Its interactions with other immune cells in TME, such as dendritic cells (DC) and T cells, are crucial to enhance the overall immune response against the tumor [28, 29]. Therefore, increased numbers or elevated functionality of NK cells contribute to better responses to ICIs [29, 30]. As for macrophages in TME, the M1/M2 macrophage paradigm plays a key role in tumor progression. M1 macrophages are commonly considered as antitumor cells, while M2 macrophages, deemed tumor-associated macrophages (TAMs), are regarded as protumor cells through angiogenic, immune suppression, hypoxia induction, and metastasis [31–33]. In addition to tumor cells, the increased expression of PDL1 of intratumoral M2 macrophages is reported to be associated with the better efficacy of ICIs in lung cancer [34]. Bioinformatics analysis has proved that M1 macrophages are needed for the efficacy of *α*PD1/*α*PDL1 therapy, which implies better treatment outcomes combined with *α*PD1/*α*PDL1 therapy [35]. Supported by the notions above, the elevated number of NK cells, the alteration of M1 and M2, and the increased level of PDL1+M2 macrophage in tumor regions implied better efficacy to some extent in our study when anlo combined with ICIs for CRC treatment.

**Figure 8:**
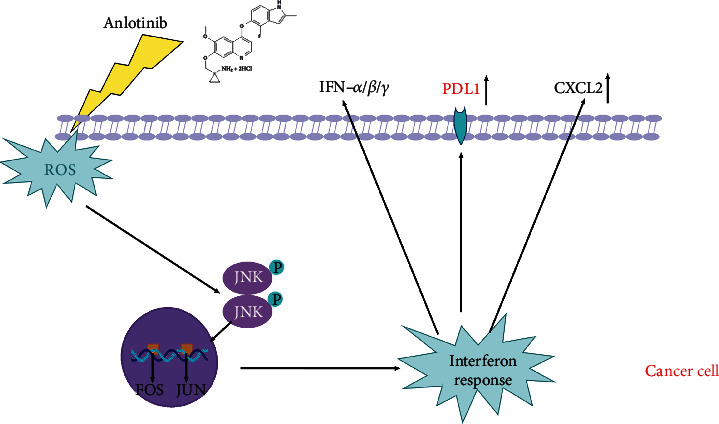
Schematic diagram of anlo-induced PDL1, IFN-*α*/*β*/*γ*, and CXCL2 expression in mouse CRC cells. Anlo increased ROS, leading to activate JNK/AP-1/interferon signaling pathway. Meanwhile, AP-1/interferons contributed to the upregulation of PDL1, INF-*α*/*β*/*γ*, and CXCL2.

ROS plays a crucial role in cell apoptosis, and we proved ROS level was elevated by the cytotoxic effects of anlo, indicating that anlo could disrupt intracellular redox homeostasis and induce oxidative stress. The c-fos and c-jun encode nuclear proteins forming a complex (AP-1) that recognizes a specific DNA sequence within the promoter region of some cellular genes [36, 37]. The DNA-binding activity of c-fos/c-jun heterodimers leads to the transcriptional regulation of target genes [38, 39]. In our current study, we verified that anlo could activate JNK/AP-1 signaling pathway which promoted the expression of PDL1, IFN-*α*/*β*/*γ*, and CXCL2 (Figure 8). Recent studies showed that transcription factors AP-1-related signal pathways could enhance the expression of many genes including PDL1, IFN-*α*/*β*/*γ*, and CXCL2, which was similar to our results above [40–43]. Moreover, some research reported that the increased level of IFN-*α*/*β*/*γ* (type I and II interferons) could upregulate the PDL1 expression [44, 45]. Therefore, the elevated PDL1 expression after anlo treatment in our research might result from both AP-1 and IFN-*α*/*β*/*γ*. According to previous research, we can find that CXCL2 is a critical regulator of neutrophil infiltration and stimulated-angiogenesis, which are favorable of immune cell infiltration [46–48]. Type I interferons (IFN-*α*/*β*) could improve the immune microenvironment by activating NK+ and T cells [49, 50]. At the same time, type II interferon (IFN-*γ*) could not only increase the expression of PDL1 on tumor cells but also reprogram macrophages to the M1 proinflammatory phenotype based on previous studies [51, 52]. What is more, type I and II interferons (IFN-*α*/*β*/*γ*) were believed to be the critical markers predicting the success of immunotherapy [53, 54]. We speculated the upregulated levels of IFN-*α*/*β*/*γ* and CXCL2 might be the reason for the increased expression of PDL1, NK cells, CD4+T cells, and M1-type macrophage cells, which may benefit the efficacy of ICIs and warrants further investigation.

In summary, our study revealed that anlo could induce intracellular oxidative stress and enhance ROS level to activate the JNK/AP-1 pathway to upregulate the expression of PDL1, IFN-*α*/*β*/*γ*, and CXCL2, which might contribute to the upregulated levels of NK cells and M1 macrophage cells. Moreover, we proved that there was a synergistic therapeutic effect when anlo combined with *α*PDL1. Finally, we found it could also elevate the PDL1 expression in human cell lines, which suggested anlo had the same elevated-PDL1 expression effect on human CRC cell lines. Therefore, we thought our study could help identify an effective therapeutic prediction for the combined treatment of anlo and *α*PDL1 therapy in clinical.

## Data Availability

The original contributions presented in the study are included in the article/supplementary materials; further inquiries can be directed to the corresponding author.

## Supplementary Materials

Supplementary Materials Supplementary table 1 shows that the primer sequences of genes for RT-PCR. Supplementary figure 1 presents the effect of *α*PDL1 on MC38/CT26 tumor cells *in vitro.* Gating strategy of T and NK+ cells and the effect of anlo on the function of NK+ cells is shown in supplementary figures 2 and 3. Supplementary figures 4 and 5 elucidate the effects of anlo on the number and function of CD4+ T and CD8+ T cells and PD1/PDL1 expression of macrophage cells in MC38 and CT26 mouse models. Supplementary figure 6 shows the effect of anlo on the expression of CXCL2 in mRNA level and cell culture serum and the expression levels of CXCL2, IFN-*β*, IFN-*γ*, and PDL1 after usage of JNK inhibitor.
