# Supplementary material for: Anlotinib Benefits the αPDL1 Immunotherapy by Activating ROS/JNK/AP-1 Pathway to Upregulate PDL1 Expression in Colorectal Cancer
Source: Oxid Med Cell Longev. 2022 Oct 4;2022:8965903. doi: 10.1155/2022/8965903 (PMC9553391; doi:10.1155/2022/8965903)
Supplement: Supplementary Materials — Supplementary table 1 shows that the primer sequences of genes for RT-PCR. Supplementary figure 1 presents the effect of αPDL1 on MC38/CT26 tumor cells in vitro. Gating strategy of T and NK+ cells and the effect of anlo on the function of NK+ cells is shown in supplementary figures 2 and 3. Supplementary figures 4 and 5 elucidate the effects of anlo on the number and function of CD4+ T and CD8+ T cells and PD1/PDL1 expression of macrophage cells in MC38 and CT26 mouse models. Supplementary figure 6 shows the effect of anlo on the expression of CXCL2 in mRNA level and cell culture serum and the expression levels of CXCL2, IFN-β, IFN-γ, and PDL1 after usage of JNK inhibitor. [file 8965903.f1.zip › supplementary Anlo--with figures.docx]

Supplementary Table 1

Primer sequences of genes for RT-PCR

| Genes | Primer sequence (5’-3’) |
| --- | --- |
| PDL1(human)  PDL1(mouse)  IFN-α  IFN-β1  IFN-γ  CXCL2  β-actin | F: TGGCATTTGCTGAACGCATTT  R: TGCAGCCAGGTCTAATTGTTTT  F: GCTCCAAAGGACTTGTACGTG  R: TGATCTGAAGGGCAGCATTTC  F: TGATGAGCTACTACTGGTCAGC  R: GATCTCTTAGCACAAGGATGGC  F: CAGCTCCAAGAAAGGACGAAC  R: GGCAGTGTAACTCTTCTGCAT  F: ATGAACGCTACACACTGCATC  R: CCATCCTTTTGCCAGTTCCTC  F: CCAACCACCAGGCTACAGG  R: GCGTCACACTCAAGCTCTG  F: GGCTGTATTCCCCTCCATCG  R: CCAGTTGGTAACAATGCCATGT |


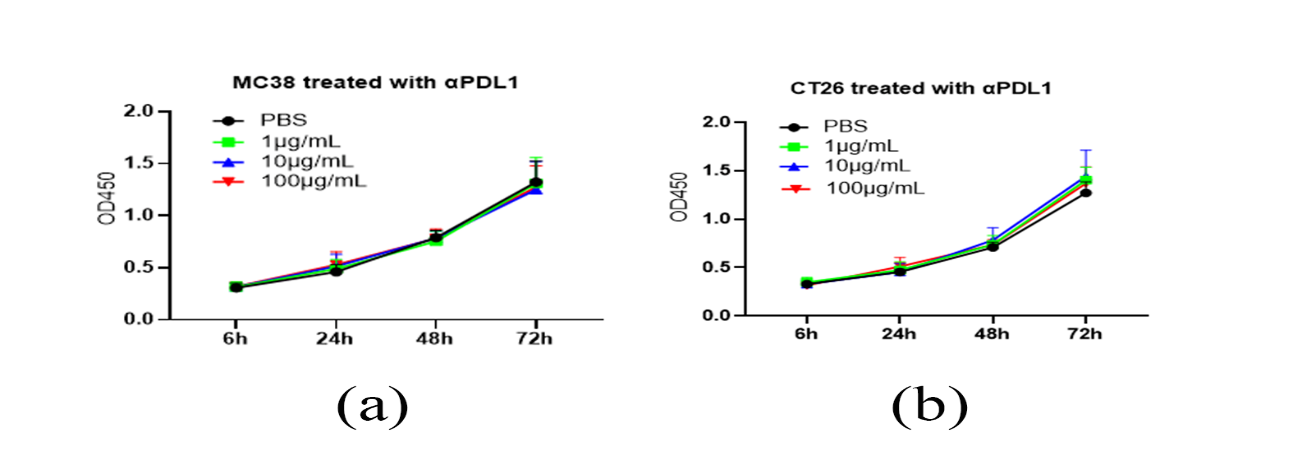


Supplementary Figure 1: The effect of αPDL1 on MC38/CT26 tumor cells *in vitro*. (a) MC38 cells were treated with different concentrations anti-PDL1(0, 1, 10, 100 μg/mL) for 6 to 72h. (b) CT26 cells were treated with different concentrations αPDL1 (0, 1, 10, 100 μg/mL) for 6 to 72h.


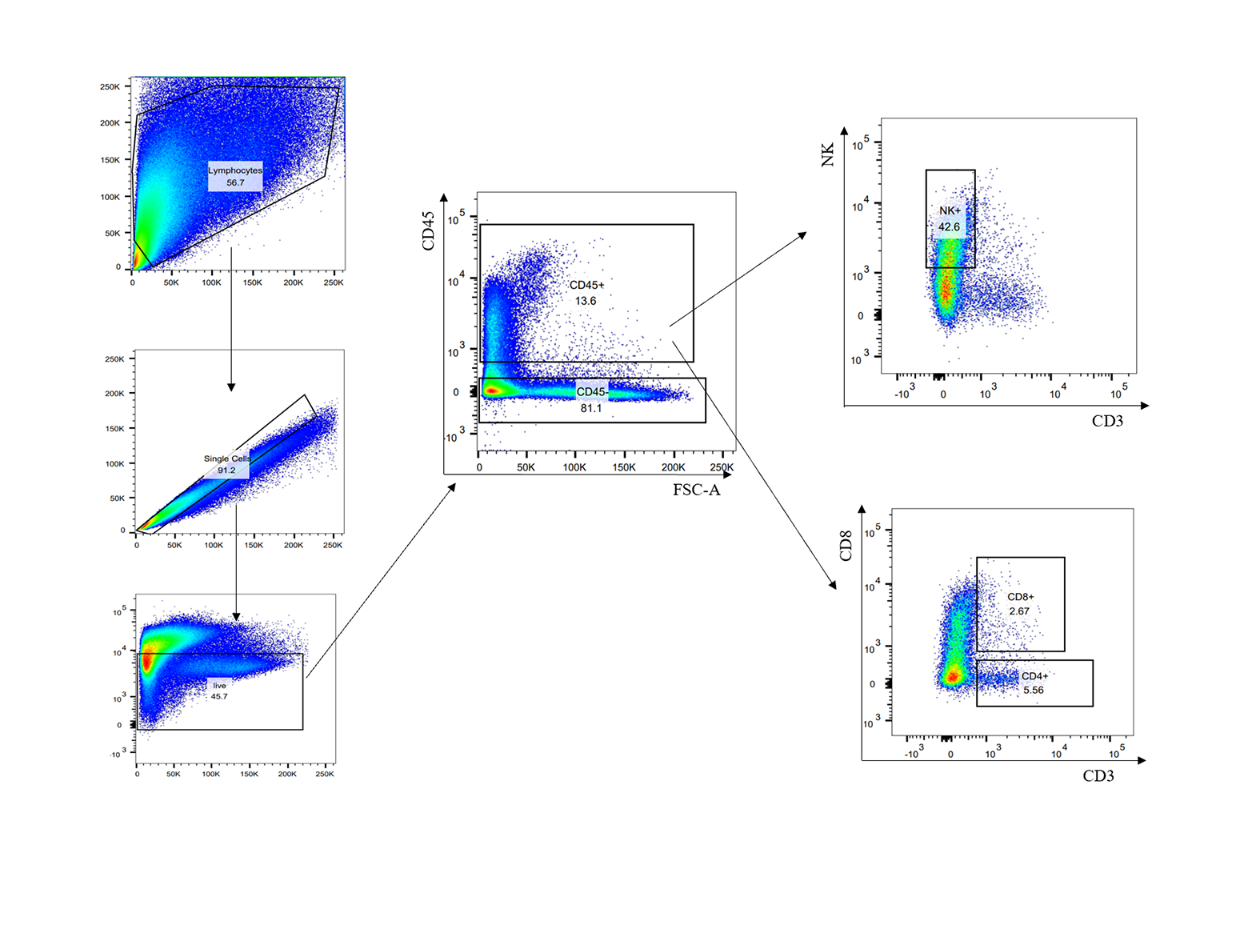


Supplementary Figure 2: Gating strategy of T and NK+ cells.


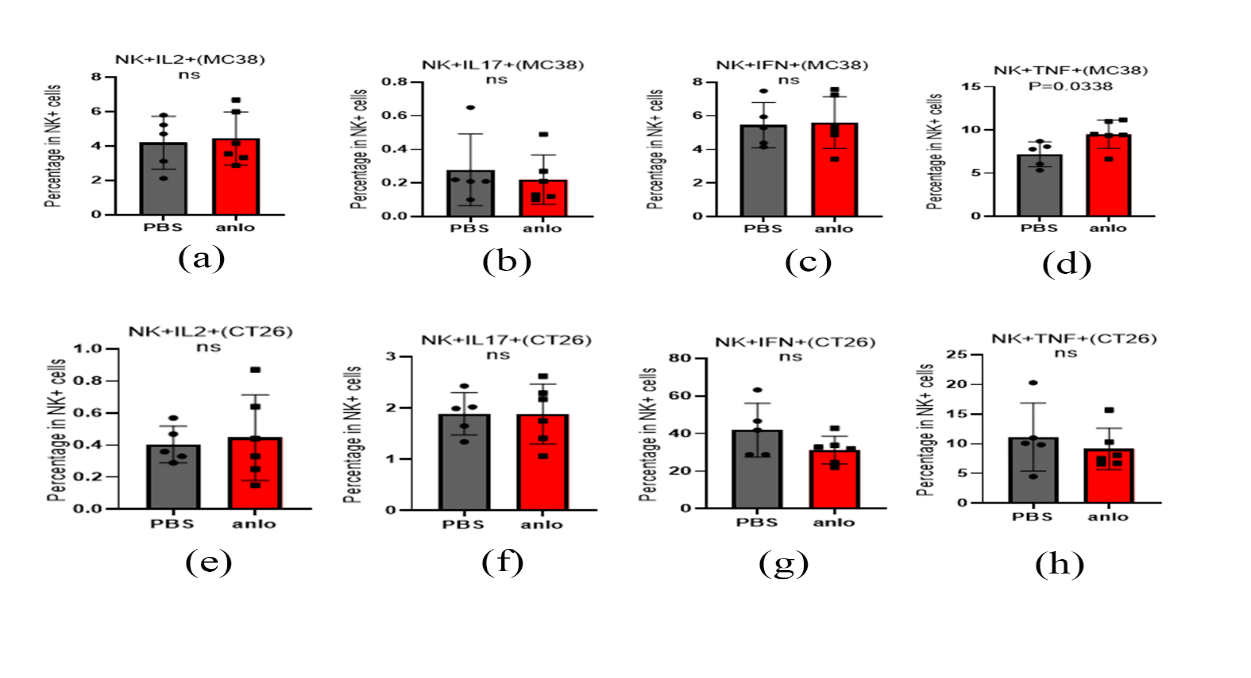


Supplementary Figure 3: The effect of anlo on the function of NK+ cells(a-h).


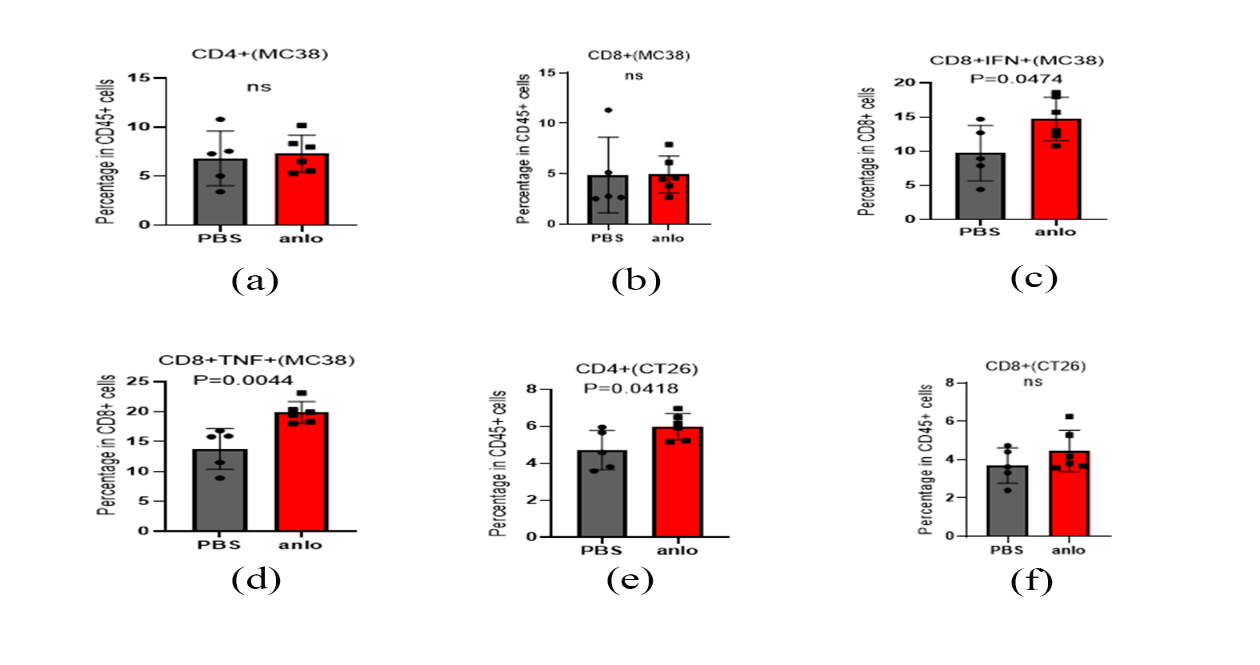


Supplementary Figure 4: The effect of anlo on the number of MC38 tumor-infiltrating CD4+ cells (a) and CD8+ cells (b), expression of IFN-γ (c) and TNF-α (d) in MC38 tumor-infiltrating CD8+ cells, the number of CT26 tumor-infiltrating CD4+ cells (e) and CD8+ cells (f).


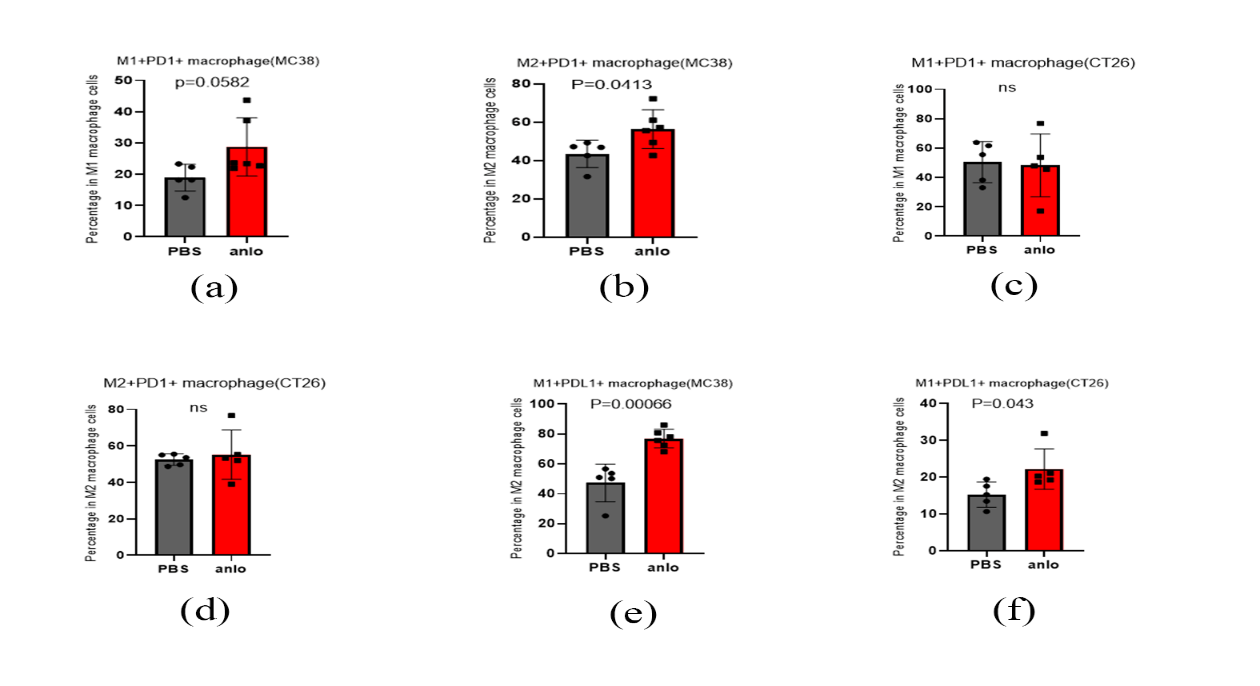


Supplementary Figure 5: The effect of anlo on the expression of PD1 in MC38 tumor-infiltrating M1 (a) and M2 cells (b), CT26 tumor-infiltrating M1 (c) and M2 cells (d), the expression of PDL1 in both MC38 (e) and CT26 (f) tumor-infiltrating M2 cells.


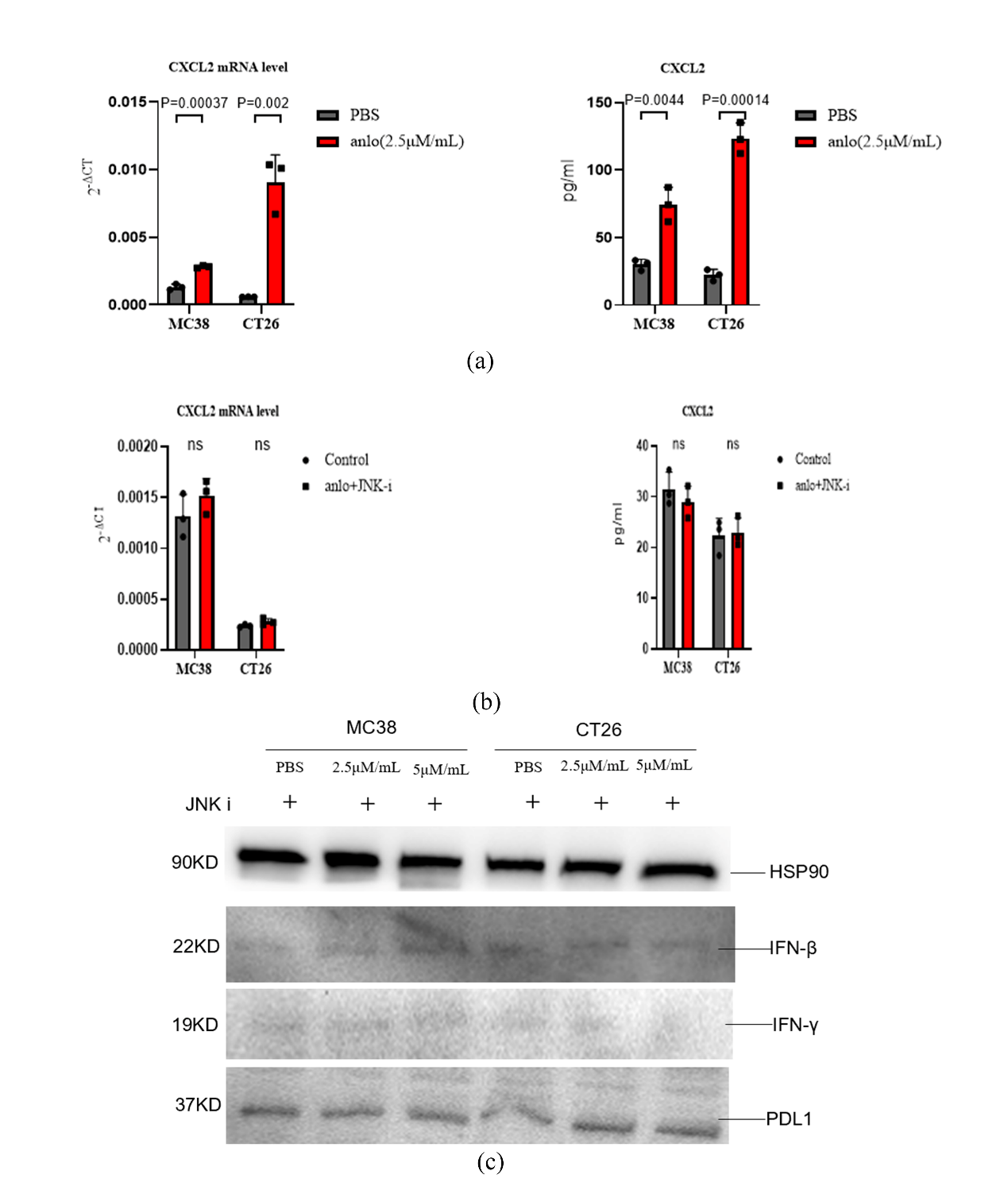


Supplementary Figure 6: The effect of anlo on the expression of CXCL2 in mRNA level and cell culture serum (a), The effect of anlo and JNK inhibitor on the expression of CXCL2 in mRNA level and cell culture serum (b), Representative western blot results of HSP90, IFN-β and IFN-γ, and PDL1 (c).
