# Supplementary figures and images for: Anlotinib Benefits the αPDL1 Immunotherapy by Activating ROS/JNK/AP-1 Pathway to Upregulate PDL1 Expression in Colorectal Cancer
Source: Oxid Med Cell Longev. 2022 Oct 4;2022:8965903. doi: 10.1155/2022/8965903 (PMC9553391; doi:10.1155/2022/8965903)

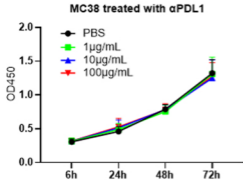

(a)

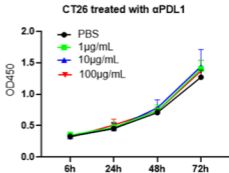

(b)

Supplement: Supplementary Materials — Supplementary table 1 shows that the primer sequences of genes for RT-PCR. Supplementary figure 1 presents the effect of αPDL1 on MC38/CT26 tumor cells in vitro. Gating strategy of T and NK+ cells and the effect of anlo on the function of NK+ cells is shown in supplementary figures 2 and 3. Supplementary figures 4 and 5 elucidate the effects of anlo on the number and function of CD4+ T and CD8+ T cells and PD1/PDL1 expression of macrophage cells in MC38 and CT26 mouse models. Supplementary figure 6 shows the effect of anlo on the expression of CXCL2 in mRNA level and cell culture serum and the expression levels of CXCL2, IFN-β, IFN-γ, and PDL1 after usage of JNK inhibitor. [file 8965903.f1.zip › figure-S1.pdf]

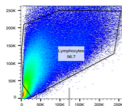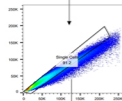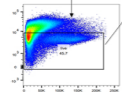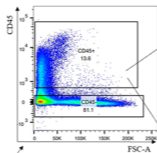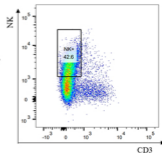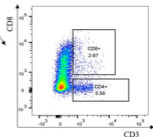

Supplement: Supplementary Materials — Supplementary table 1 shows that the primer sequences of genes for RT-PCR. Supplementary figure 1 presents the effect of αPDL1 on MC38/CT26 tumor cells in vitro. Gating strategy of T and NK+ cells and the effect of anlo on the function of NK+ cells is shown in supplementary figures 2 and 3. Supplementary figures 4 and 5 elucidate the effects of anlo on the number and function of CD4+ T and CD8+ T cells and PD1/PDL1 expression of macrophage cells in MC38 and CT26 mouse models. Supplementary figure 6 shows the effect of anlo on the expression of CXCL2 in mRNA level and cell culture serum and the expression levels of CXCL2, IFN-β, IFN-γ, and PDL1 after usage of JNK inhibitor. [file 8965903.f1.zip › figure-S2.pdf]

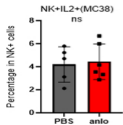

(a)

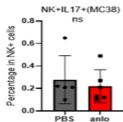

(b)

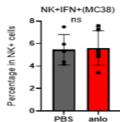

(c)

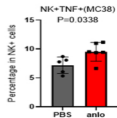

(d)

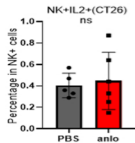

(e)

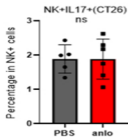

(f)

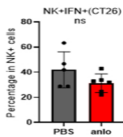

(g)

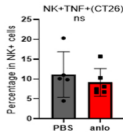

(h)

Supplement: Supplementary Materials — Supplementary table 1 shows that the primer sequences of genes for RT-PCR. Supplementary figure 1 presents the effect of αPDL1 on MC38/CT26 tumor cells in vitro. Gating strategy of T and NK+ cells and the effect of anlo on the function of NK+ cells is shown in supplementary figures 2 and 3. Supplementary figures 4 and 5 elucidate the effects of anlo on the number and function of CD4+ T and CD8+ T cells and PD1/PDL1 expression of macrophage cells in MC38 and CT26 mouse models. Supplementary figure 6 shows the effect of anlo on the expression of CXCL2 in mRNA level and cell culture serum and the expression levels of CXCL2, IFN-β, IFN-γ, and PDL1 after usage of JNK inhibitor. [file 8965903.f1.zip › figure-S3.pdf]

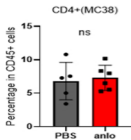

(a)

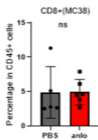

(b)

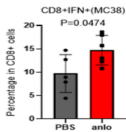

(c)

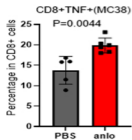

(d)

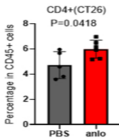

(e)

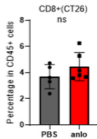

(f)

Supplement: Supplementary Materials — Supplementary table 1 shows that the primer sequences of genes for RT-PCR. Supplementary figure 1 presents the effect of αPDL1 on MC38/CT26 tumor cells in vitro. Gating strategy of T and NK+ cells and the effect of anlo on the function of NK+ cells is shown in supplementary figures 2 and 3. Supplementary figures 4 and 5 elucidate the effects of anlo on the number and function of CD4+ T and CD8+ T cells and PD1/PDL1 expression of macrophage cells in MC38 and CT26 mouse models. Supplementary figure 6 shows the effect of anlo on the expression of CXCL2 in mRNA level and cell culture serum and the expression levels of CXCL2, IFN-β, IFN-γ, and PDL1 after usage of JNK inhibitor. [file 8965903.f1.zip › figure-S4.pdf]

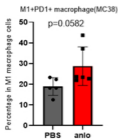

(a)

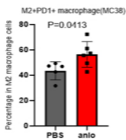

(b)

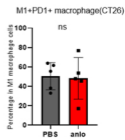

(c)

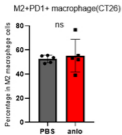

(d)

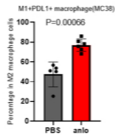

(e)

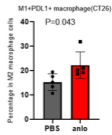

(f)

Supplement: Supplementary Materials — Supplementary table 1 shows that the primer sequences of genes for RT-PCR. Supplementary figure 1 presents the effect of αPDL1 on MC38/CT26 tumor cells in vitro. Gating strategy of T and NK+ cells and the effect of anlo on the function of NK+ cells is shown in supplementary figures 2 and 3. Supplementary figures 4 and 5 elucidate the effects of anlo on the number and function of CD4+ T and CD8+ T cells and PD1/PDL1 expression of macrophage cells in MC38 and CT26 mouse models. Supplementary figure 6 shows the effect of anlo on the expression of CXCL2 in mRNA level and cell culture serum and the expression levels of CXCL2, IFN-β, IFN-γ, and PDL1 after usage of JNK inhibitor. [file 8965903.f1.zip › figure-S5.pdf]

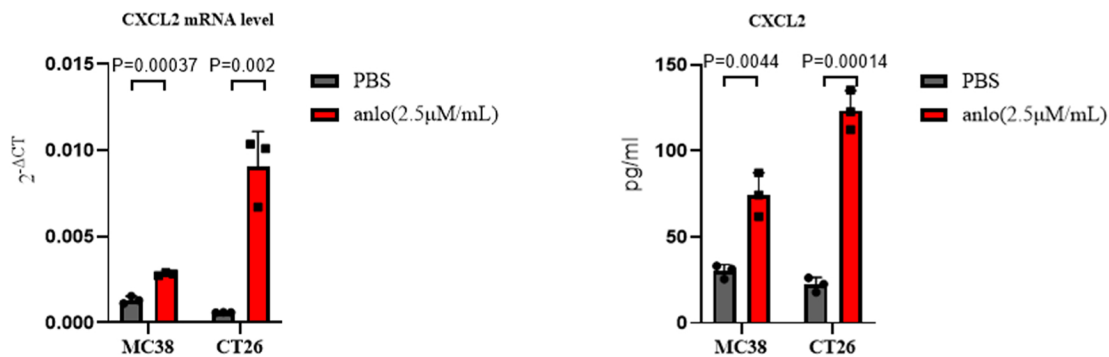

(a)

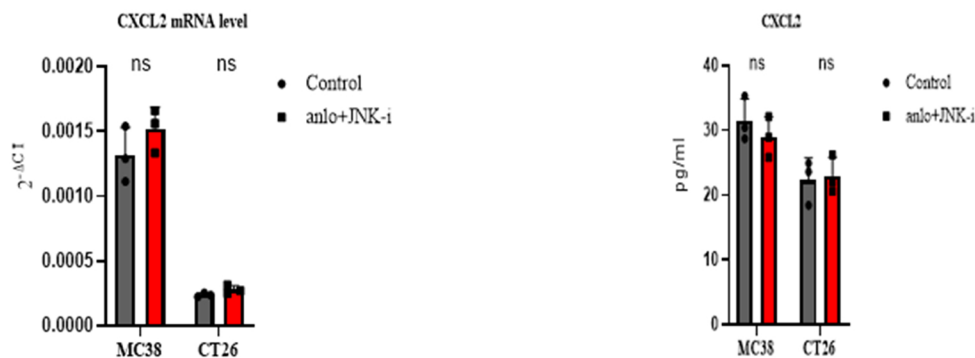

(b)

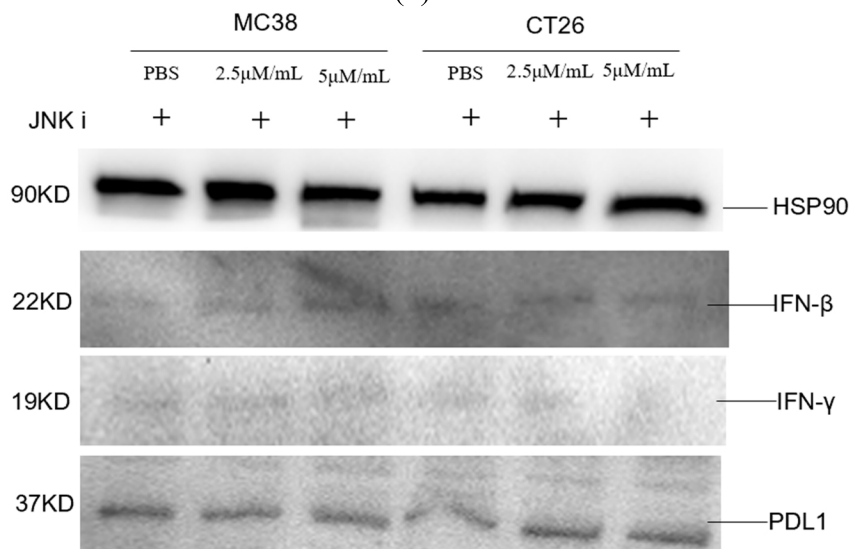

(c)

Supplement: Supplementary Materials — Supplementary table 1 shows that the primer sequences of genes for RT-PCR. Supplementary figure 1 presents the effect of αPDL1 on MC38/CT26 tumor cells in vitro. Gating strategy of T and NK+ cells and the effect of anlo on the function of NK+ cells is shown in supplementary figures 2 and 3. Supplementary figures 4 and 5 elucidate the effects of anlo on the number and function of CD4+ T and CD8+ T cells and PD1/PDL1 expression of macrophage cells in MC38 and CT26 mouse models. Supplementary figure 6 shows the effect of anlo on the expression of CXCL2 in mRNA level and cell culture serum and the expression levels of CXCL2, IFN-β, IFN-γ, and PDL1 after usage of JNK inhibitor. [file 8965903.f1.zip › figure-S6.pdf]
